# Corophiine amphipods of the genera *Chelicorophium* and *Paracorophium* from the lower Gulf of Thailand (Crustacea, Amphipoda, Corophiidae, Corophiinae)

**DOI:** 10.3897/zookeys.505.9751

**Published:** 2015-05-21

**Authors:** Koraon Wongkamhaeng, Jaruwat Nabhitabhata, Prawit Towatana

**Affiliations:** 1 Marine and Coastal Resources Institute (MACORIN), Prince of Songkla University, 90112 Thailand; 2 Excellence Center for Biodiversity of Peninsular Thailand, Faculty of Science, Prince of Songkla University, 90112 Thailand

**Keywords:** Crustacea, Amphipoda, new species, taxonomy, Thai waters

## Abstract

Two species of corophiine amphipods from Songkhla Lake, in the lower Gulf of Thailand, are described and illustrated. *Chelicorophium madrasensis* (Nayar, 1950), found in the mangrove forest, has not previously been observed in Thai waters. *Paracorophium angsupanichae* **sp. n.** is characterized by its chelate male gnathopod 2, obtuse palm with subrectangular distomedial elevation, and urosomites 1-3 free. This is the first record of the genus *Chelicorophium* and *Paracorophium* in Thai waters. All specimens are deposited in the Princess Maha Chakri Sirindhorn Natural History Museum, Prince of Songkla University, Thailand and the Museum für Naturkunde, Berlin.

## Introduction

The subfamily Corophiinae was established by Bousfield and Hoover in 1997 and is defined by its gnathopods 1 and 2, together forming a sieving structure with dense sieving setae on the posterior margins of the carpus and ischium. Corophiinae are world wide distributed, most of them living in brackish or freshwater (Myers 2009). In Thai waters, only *Monocorophium acherusicum* (Costa, 1853) has been recorded in Songkhla Lake, the largest natural lagoon located in Southern Thailand (Angsupanich and Kuwabara 1995). This study focuses on the hitherto poorly known gammarid amphipods in the Gulf of Thailand. The first is a new corophiine species of *Paracorophium angsupanichae*, which was found in Songkhla Lake. The second is *Chelicorophium madrasensis* (Nayar, 1950), which has not been previously recorded in Thai waters. Figures and descriptions of both species are provided.

## Material and methods

**Figure 1.**
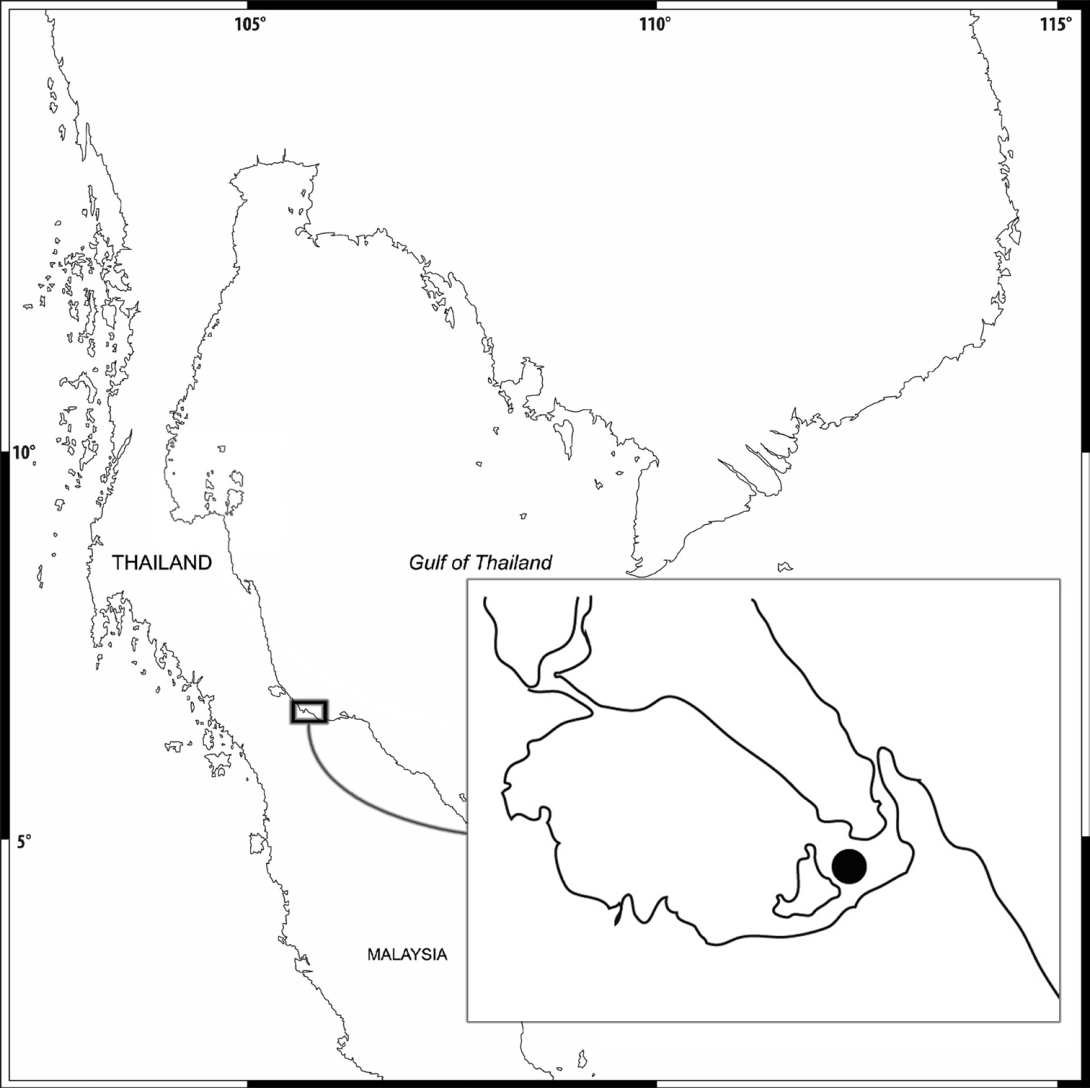
Map of the sampling area.

Amphipods were collected from Songkhla Lake (Figure 1). The sites were visited at low tide, and amphipods were collected using a 20 × 20 cm Ekman grab. The amphipod specimens were sorted and fixed in formalin for one week and then stored in 70% alcohol. In the laboratory, the specimens were transferred from alcohol into glycerol for study. The drawings of body parts were accomplished using a drawing tube attached to an Olympus CH30 light microscope. The pencil drawings were scanned and digitally inked using a WACOM bamboo CTH-970 graphics board, following the method described in Coleman (2003). The following abbreviations are used: A, antenna; G, gnathopod; HD, head; LL, lower lip; MD, mandible; MX, maxilla; MP, maxilliped; P, pereopod; Pl, pleopod; T, telson; U, uropod; UR, urosome; UL, upper lip; r, right; l, left; ♂, male; and ♀, female. Specimens of different species were deposited in the Prince of Songkla University Zoological Collection (PSUZC) and the Museum für Naturkunde, Berlin (ZMB).

## Results

### Systematics
Corophiidae Leach, 1814

#### Corophiinae

Taxon classificationAnimaliaAmphipodaCorophiidae

Leach, 1814

Paracorophium Stebbing, 1899.

##### Diagnosis.

Labrum symmetrically incised, labium normal,with innerlobes. Maxilla 1: inner lobe small, palp 2-articulate. Maxilliped: inner margin of outer lobe with several slender spines. Mandible: molar triturative, incisor toothed, palp 3-articulate. Rostrum short, coxa 4 without distoposterior lobe, coxa 5 as long as coxa 4. Accessory flagellum absent. Gnathopod 1 subchelate, gnathopod 2 merochelate, distally chelate, parachelate or subchelate. Basis of pereopods 5-7 not lobed. Peduncle of uropod 1 with distoventral strong tooth. Uropod 3 very short, biramous, rami unisegmented. Telson short, fleshy, entire, bearing 2 distal corner teeth. Oostegyts narrow, coxal gills simple. Sexual dimorphism present (gnathopod 2).

##### Type species.

*Corophium excavatum* Thomson, 1884 (type by monotypy)

##### Species composition.

*Paracorophium excavatum* (G.M. Thomson, 1884); *Paracorophium lucasi* Hurley, 1954; *Paracorophium chelatum* (G. Karaman, 1979); *Paracorophium hartmannorum* Andres, 1979; *Paracorophium chilensis* Varela, 1983; *Paracorophium brisbanensis* Chapman, 2002; *Paracorophium nana* Myers, 2009. *Paracorophium angsupanichae* sp. n. (this study).

#### Paracorophium angsupanichae sp. n.

Taxon classificationAnimaliaAmphipodaCorophiidae

http://zoobank.org/172494B3-14BB-4487-A9FA-1EB9DFEC1E66

**Figure 2.**
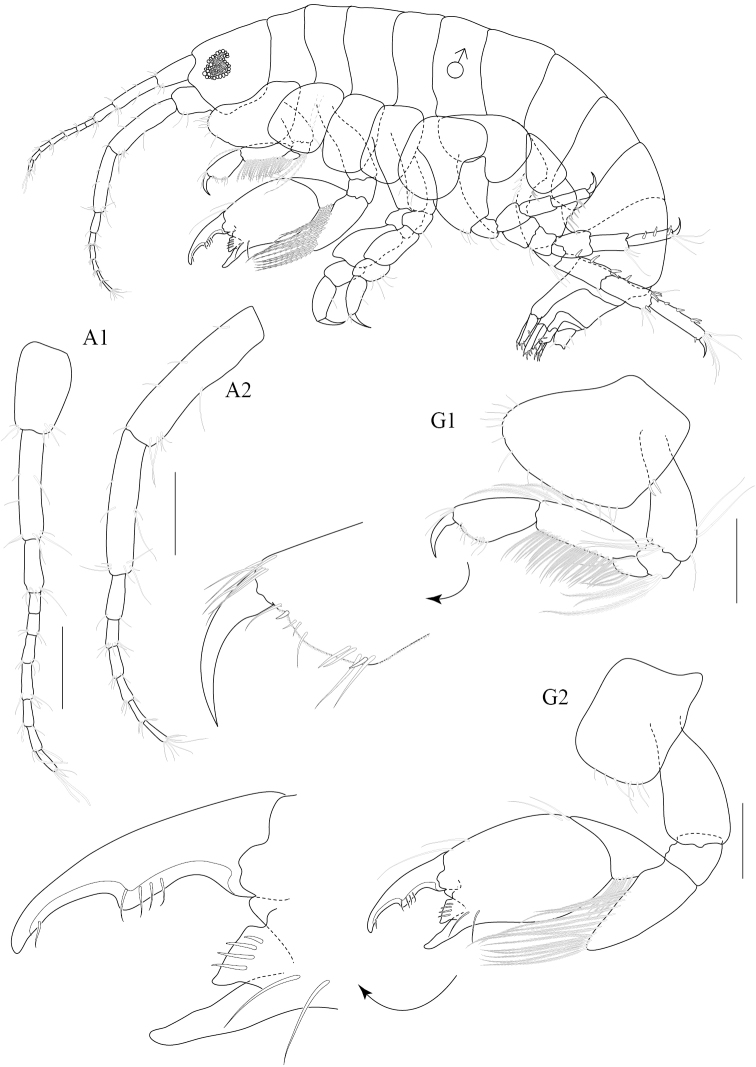
*Paracorophium angsupanichae* sp. n. holotype, male (PSUZC-CR-0350), 1.75 mm. Middle Songkhla Lake, Lower Gulf of Thailand. All scale bars represent 0.2 mm.

**Figure 3.**
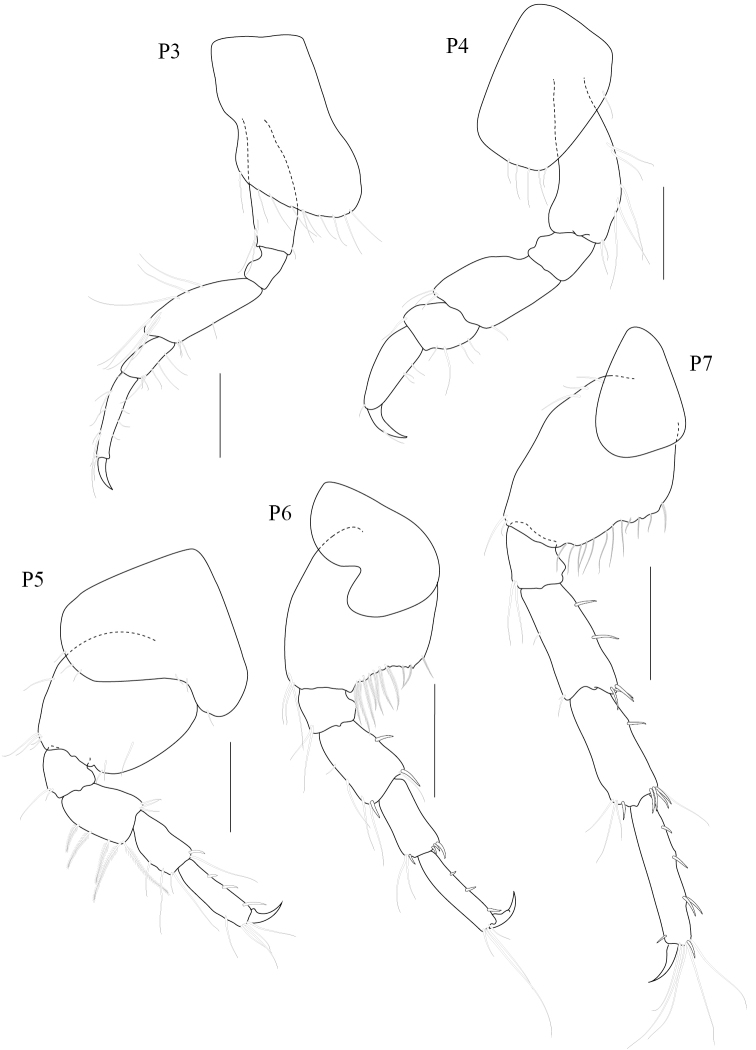
*Paracorophium angsupanichae* sp. n. holotype, male (PSUZC-CR-0350), Middle Songkhla Lake, Lower Gulf of Thailand. The scale bars for P3-5 represent 0.2 mm and scale bars for P6-7 represent 0.5 mm.

**Figure 4.**
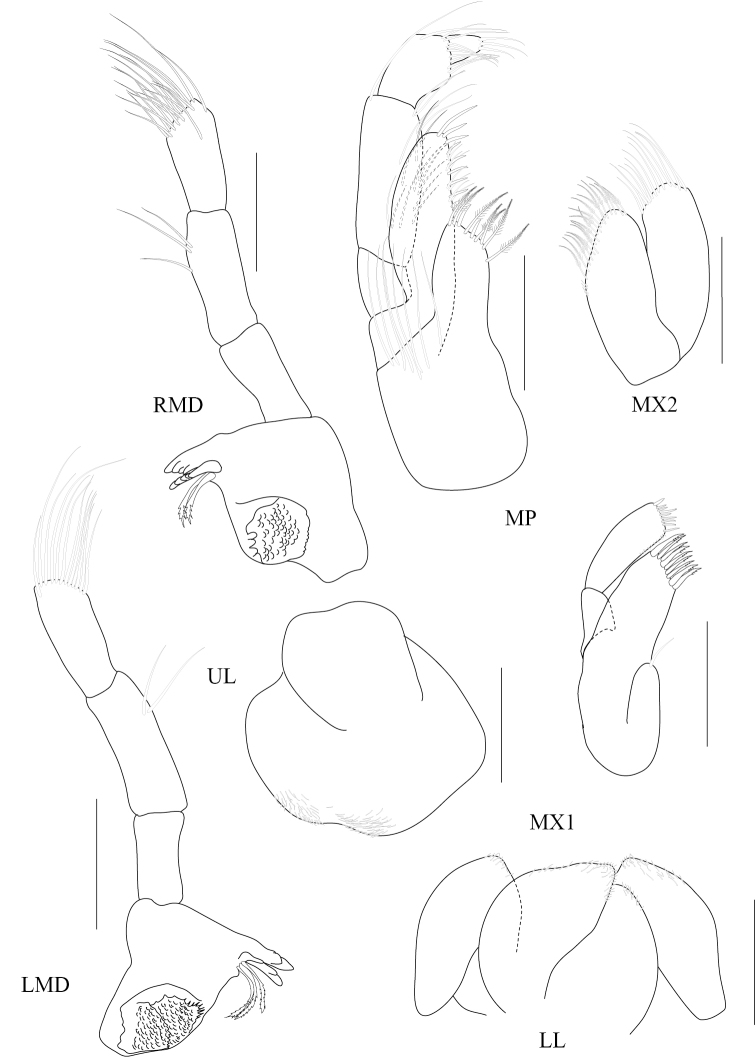
*Paracorophium angsupanichae* sp. n. holotype, male (PSUZC-CR-0350), Middle Songkhla Lake, Lower Gulf of Thailand. All scale bars represent 0.1 mm.

**Figure 5.**
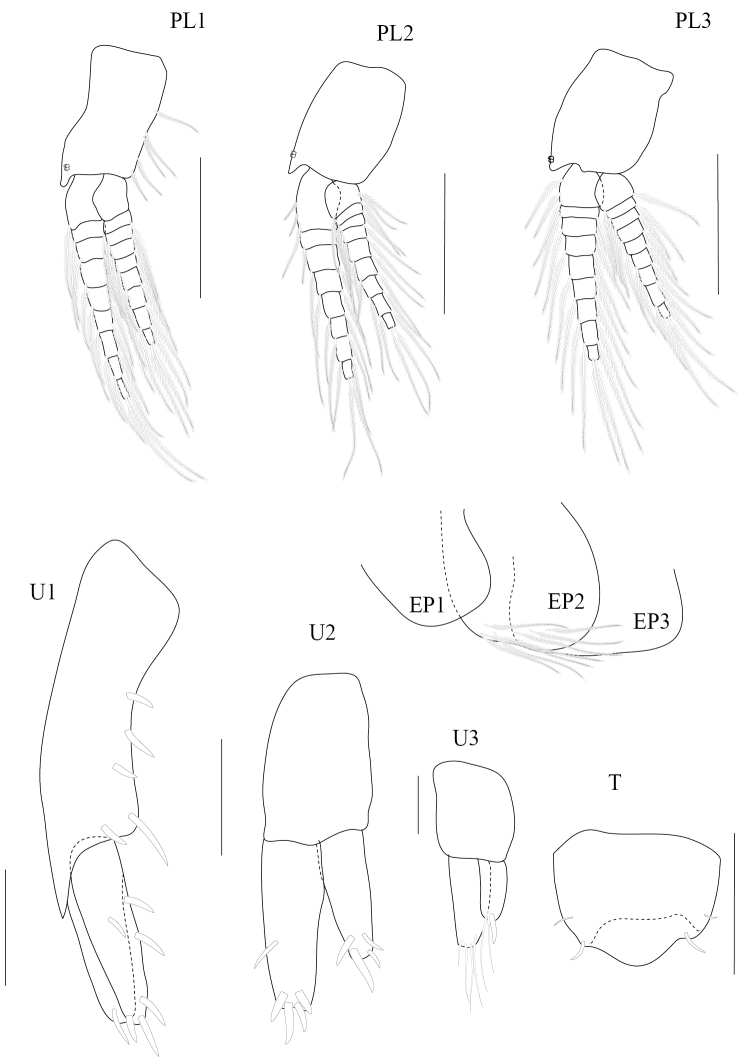
*Paracorophium angsupanichae* sp. n. holotype, male (PSUZC-CR-0350), Middle Songkhla Lake, Lower Gulf of Thailand. The scale bars for U1-U3, PL1-3 represent 0.2 mm, but 0.1 mm for T.

**Figure 6.**
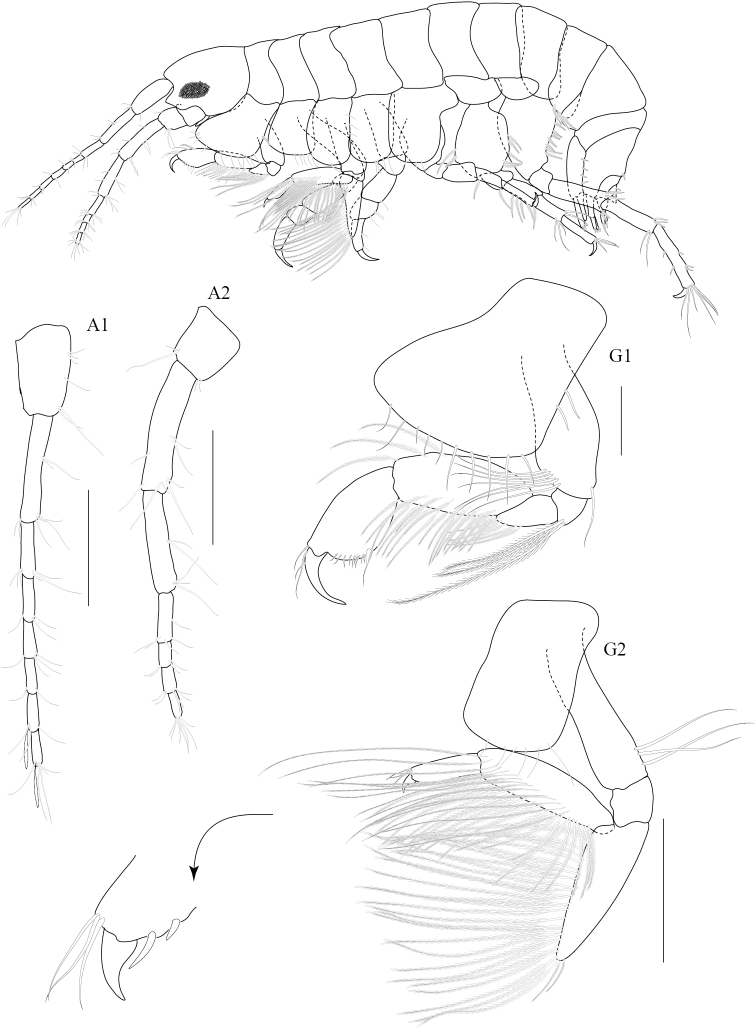
*Paracorophium angsupanichae* sp. n. allotype, female (PSUZC-CR-0351), 2.6 mm, Middle Songkhla Lake, Lower Gulf of Thailand. All scale bars represent 0.2 mm.

Figs 2 , 3 , 4 , 5 , 6

##### Material examined.

Holotype. ♂, THAILAND, Lower Gulf of Thailand, Middle Songkhla Lake (7°28'36"N, 100°24'6"E), 23 October 2014, leg. K. Wongkamhaeng, PSUZC-CR-0350. Allotype, ♀ collected with holotype; PSUZC-CR-0351; Paratypes, collected with holotype PSUZC-CR-0352 (5♂; 5♀) and ZMB28473 (3 ♂;3♀)

##### Description.

Based on male holotype. Body length 1.75 mm (from tip of rostrum to apex of telson). *Body* compressed, smooth, urosomites 1-3 free. *Head*, lateral cephalic lobe rounded. *Antenna 1* sparsely setose, short, one third of body length, ratios of peduncular articles 1—3 5:6:3; accessory flagellum absent; primary flagellum 7-articulate, bearing 4-6 aesthetascs. *Antenna 2*, sparsely setose; flagellum 6-articulate. *Upper lip* (labrum) symmetrically incised distally. *Lower lip* (labium), inner lobes well developed. *Mandible*, both similar, left incisor 3-dentate, right incisor 4-dentate; left and right lacinia mobilis armed with 3 and 4 teeth respectively; ratios of palp article 1-3 5:8:6. *Maxilla 1*, inner plate short; outer plate with 9 bifid robust setae; palp 2-articulate, second article with 9 distal robust setae. *Maxilla 2*, inner lobe with distal and marginal plumose setae; outer lobe with 13 fine setae. *Maxilliped*, inner plate exceeding palp article 1, oblique, with 8 basal setae, 7 apical plumose setae and 2 apical robust setae; outer plate not reaching apex of palp article 2, with 9 marginal robust setae and 2 fine apical setae; palp 4-articulate; ratios of peduncular articles 1-4 3:7:4:2.

**Pereon.** *Gnathopod 1* subchelate, smaller than gnathopod 2; coxal plate trapezium-shaped, produced anteriorly with 7 fine setae on anteroventral corner and 2 robust setae on posterior margin; length ratio of articles from basis to dactylus 3:1:1:3:2:1; basis slender, broader distally, with anterodistal setae and posteromarginal setae; ischium short, subrectangular; merus subtriangular with posteromarginal setae; carpus long, with plumose setae on both margins; propodus subtriangular, palm oblique with a defining robust seta. *Gnathopod 2* chelate; coxal plate subquadrate; length ratio of articles from basis to dactylus 8:3:7:6:12:6; basis robust, expanded distally; merus subtriangular, distal angle produced with a group of very long plumose setae on anterior margin; carpus medially expanded, bearing dense setae on posterior margin; propodus suboval, narrowing distally with a weak subdistal excavation on posterior margin, anterior margin sparsely setose, palm obtuse with subrectangular distomedial elevation bearing 4 robust setae; dactylus overlapping palm, expanded medially with 3 fine setae and 1 distal seta. *Pereopod 3* slender; coxal plate deeper than long, slightly expanded ventrally, beset with marginal setae; length ratio of articles from basis to dactylus 6:2:6:2:5:2; basis slender; ischium short; merus slightly produced anterodistally, anterior margin lined with 9 long setae and short posterodistal setae; carpus and propodus slender, lined with marginal setae on both sides; dactylus curved. *Pereopod 4* similar to pereopod 3; length ratio of articles from basis to dactylus 6:2:4:2:3:1; basis slender, with long fine setae on posterior margin; merus slightly produced anterodistally; carpus suboval with a weak subdistal excavation, with setae on both margins; propodus long and narrow, sparse setae; dactylus long and thin with one seta. *Pereopod 5–7* progressively longer, in the length ratio 2:3:4. *Pereopod 5* coxa bilobed; length ratio of articles from basis to dactylus 8:2:4:3:4:1; basis oval, sparsely setose; merus and carpus subequal with anteromarginal setae; propodus slender with 3 marginal robust setae; dactylus curved. *Pereopod 6* coxa bilobed; length ratio of articles from basis to dactylus 9:2:4:4:5:1; basis oval, posterodistally excavated, lined with 10 plumose setae; merus subrectangular with a median robust seta and distal setae; carpus subrectangular with distal robust setae; propodus slender with 3 marginal setae; dactylus long and curved. *Pereopod 7* coxa subtriangular; basis suboval, posterodistally excavated; merus – propodus slender with marginal and distal robust setae; dactylus tapering to pointed tip.

**Pleon.** *Pleopods 1–3* well developed; peduncles subrectangular with 2 retinaculae, longer than broad; rami unequal, inner ramus longer than outer ramus. *Epimeron* 1–3 subquadrate; epimeron 2 bearing 7 plumose setae. *Uropod 1* not extending beyond ends of other uropods; peduncle longer than rami, fringed with robust setae, peduncular apex bearing a posteroventral process; outer and inner ramus subequal, both rami lined with a row of robust setae, distal margins rounded and bearing several robust setae. *Uropod 2* peduncle subequal to rami; outer ramus slightly longer than inner one, both rami with apical robust setae and outer ramus bearing medial robust setae. *Uropod 3*, peduncle subequal to rami subequal to outer ramus, which is slightly longer than inner one, bearing 4 apical setae; inner ramus with 2 fine apical setae and 1 robust seta. *Telson* subtrapezoidal, broader than long, each distal margin with distal spine.

**Female** (*allotype*). Total body length 2.6 mm (from tip of rostrum to apex of telson). Sexually dimorphic characters: *Antenna 1* flagellum with 5 articles, last 2 articles bearing aesthetascs. *Gnathopod 2* coxa much deeper than wide; basis elongate, expanded distally; merus subtriangular with a long plumose setae on anterior margin; carpus subrectangular, bearing long plumose setae on posterior margin; propodus elongate with posterior plumose marginal setae, palm poorly developed with 2 robust setae; dactylus curved.

##### Etymology.

The species is named in honor of Professor Dr. Saowapa Angsupanich of Prince of Songkla University, Thailand, who contributed to the study of the ecosystem in Songkhla Lake.

##### Remarks.

*Paracorophium angsupanichae* sp. n. is characterized by male gnathopod 2 chelate and shares this character with *Paracorophium chelatum* in the Palau Islands, in the southern Pacific Ocean, east of the Philippines. Both amphipods inhabit freshwater environments above tidal influence. However, *Paracorophium chelatum* has the following characters (Karaman 1979): 1) its maxilliped lacks the distinctive rows of long setae on the basal segment (present in *Paracorophium angsupanichae*), 2) its male gnathopod 2 palm is smooth (palm is obtuse with subrectangular distomedial elevation bearing 4 robust setae), and 3) its urosomites 1–2 are fused (urosomites 1–3 are free in *Paracorophium angsupanichae*).

#### Chelicorophium

Taxon classificationAnimaliaAmphipodaCorophiidae

Bousfield & Hoover, 1997

##### Diagnosis.

(modified from Bousfield and Hoover 1997) Epistome produced. Lower lip, mandibular lobes medium. Mandibular palp basic (type PI of Hirayama 1987). Maxilla 1, palp sublinear, longer than outer plate. Maxilliped, inner plate short, apex subacute, inner margin with basal spine; outer plate regular, inner margin strongly setose; palp segment 2 medium to long. Rostrum short. Antenna 2 strongly pediform and well developed (clasping) in both sexes; peduncular segment 4 with strong bidentate posterodistal process; segment 5 short, usually with median tooth near mid-point. Gnathopod 1 subchelate. Gnathopod 2 propod slender, not longer than combined merus and carpus; dactyl short, typically tridentate. Urosome segments not coalsed. Uropods 1 and 2 medium, peduncles broadening distally; rami short, straight; inner and outer margins often spinose or setose, apex little out-curved. Uropod 3, ramus longer than peduncle, slightly broadened, setose apically. Telson short, broad, spinose hooks at hind comers and dorsally. Coxal gills medium broad, sac-like, on pereopods 3-6. Brood lamellae short, sublinear, marginal setae not elongate.

#### Chelicorophium madrasensis

Taxon classificationAnimaliaAmphipodaCorophiidae

Nayar, 1950

##### Material examined.

Lower Gulf of Thailand, Songkhla Lake (09°18'39.5"N, 99°46'46.4"E), mangrove forest, 1 Feb 2012, leg. R. Puttapreecha, PSUZC-CR-0353 (4♂; 5♀), ZMB28474 (2♂; 2♀)

##### Type locality.

Madras Coast, India.

##### Description.

*Body* subcylindrical; urosomites 1–3 free. *Head*, rostrum short, not exceeding anterior head lobes; inferior antennal sinus deeply regressed. *Antenna 1* slender, one third of body length; peduncle article 1 setose; primary flagellum 12-articulate. *Antenna 2* strongly developed in both sexes, much longer than antenna 1; peduncle setose along ventral margin; peduncular segment 4 with strong bidentate posterodistal process; segment 5 shorter, without median tooth; flagellum 3-articulate;

*Lower lip* (Labium), inner lobe well developed, mandibular lobes medium. *Mandible* both similar, incisor 3 dentate; left and right lacinia mobilis armed with 2 and 3 dentate respectively; first article of palp with long pilous seta. *Maxilla 1*, palp longer than outer plate. *Maxilla 2*, inner lobe shorter than outer lobe; both bearing distal and marginal plumose setae; *Maxilliped*, inner plate short, apex subacute, inner margin with marginal setae; outer plate reaching apex of palp article 2, setose along both sides; palp segments 2-4 with marginal setae.

**Pereon.** *Gnathopod 1* subchelate-rectipalmate; merus and carpus bearing posteriomarginal plumose setae. *Gnathopod 2* merus as long as carpus, with long plumose setae along posterior margin; dactylus tridentate. *Pereopods 3 and 4* alike, basis broad (glandular); dactylus subequal to propodus. *Pereopod 5* short, basis setose along posterior margin; carpus short, bearing two rows of robust setae along posterior margin. *Pereopod 6* basis sparsely setose; carpus short not elongate, bearing two rows of robust setae along posterior margin; dactyl short. *Pereopod 7* elongate; basis posteriorly strongly setose on both margins; ischium – carpus sparsely setose; propodus with marginal and distal setae.

**Pleon.** *Urosomites* 1-3 free. *Uropods 1 and 2* peduncles slightly broadening distally; rami short, straight; outer ramus beset with robust setae. *Uropod 3*, ramus subequal to peduncle, apically setose. Telson short, truncate, broader than long.

**Female.** No sexual differences.

**Figure 7.**
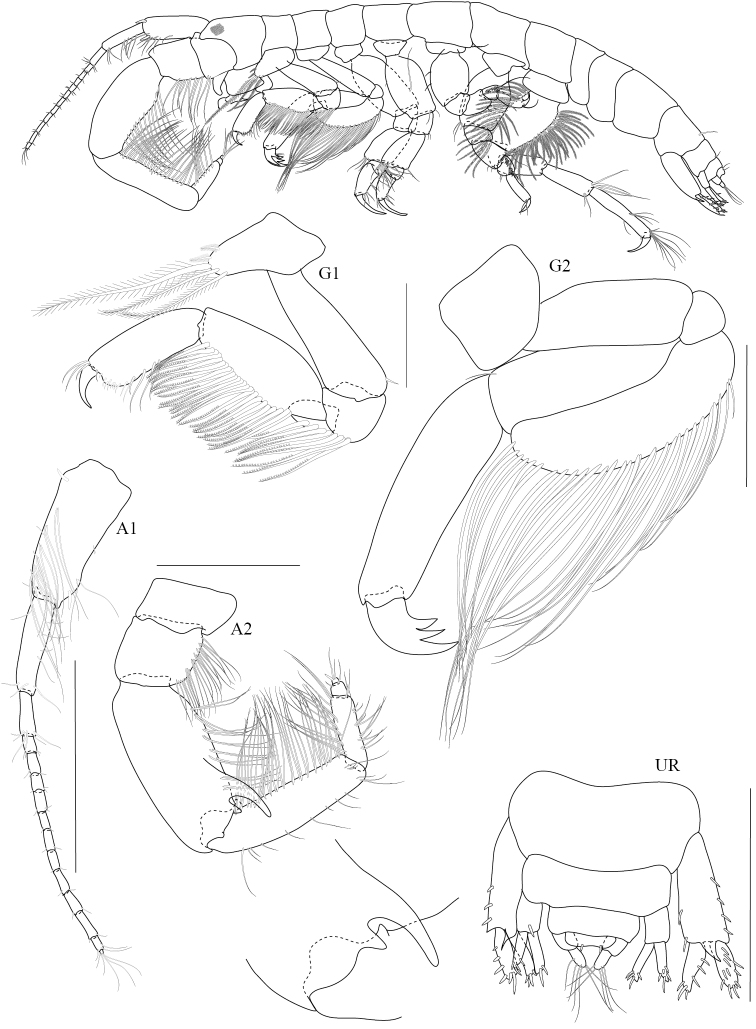
*Chelicorophium madrasensis* male (PSUZC-CR-0353), Lower Songkhla Lake, Lower Gulf of Thailand. All scale bars represent 0.2 mm.

**Figure 8.**
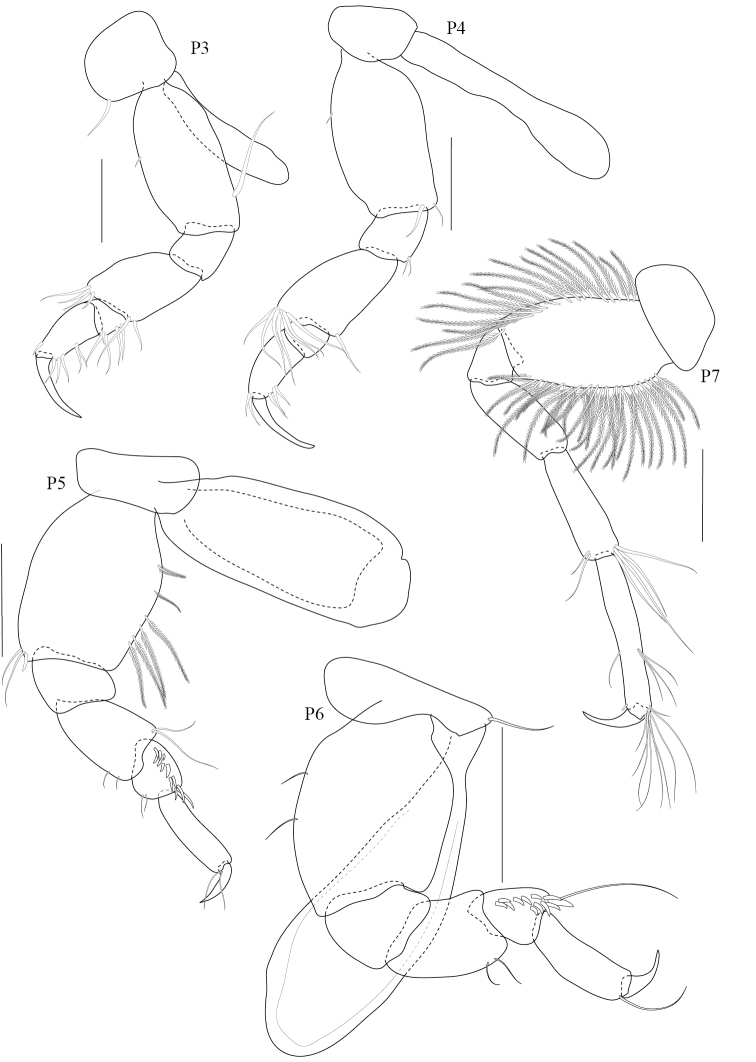
*Chelicorophium madrasensis* male (PSUZC-CR-0353), Lower Songkhla Lake, Lower Gulf of Thailand. All scale bars represent 0.2 mm.

**Figure 9.**
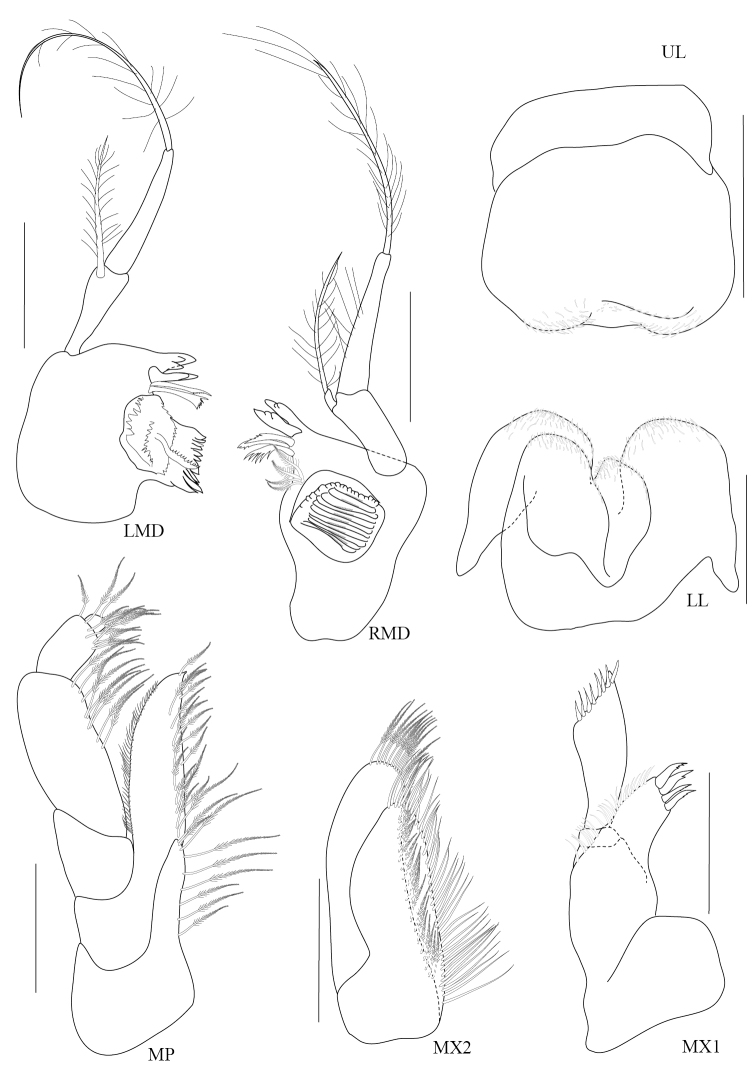
*Chelicorophium madrasensis* male (PSUZC-CR-0353), Lower Songkhla Lake, Lower Gulf of Thailand. All scale bars represent 0.1 mm.

##### Remarks.

Nayar (1950) described *Chelicorophium madrasensis* from the Madras Coast, India, which is characterized by antenna 2 article 4 inner surface with 2 proximal spines and epimeron 1 smooth and naked. The specimens from the current study are similar to those of Nayar, but the telson is truncated, whereas it is pointed in *Chelicorophium madrasensis*.

##### Distribution.

Indian Ocean and Songkhla Lake (current study).

## Supplementary Material

XML Treatment for Corophiinae

XML Treatment for Paracorophium angsupanichae

XML Treatment for Chelicorophium

XML Treatment for Chelicorophium madrasensis
